# Supplementary figures and images for: Clinical evaluation of BCL-2/XL levels pre- and post- HER2-targeted therapy
Source: PLoS One. 2021 May 5;16(5):e0251163. doi: 10.1371/journal.pone.0251163 (PMC8099090; doi:10.1371/journal.pone.0251163)

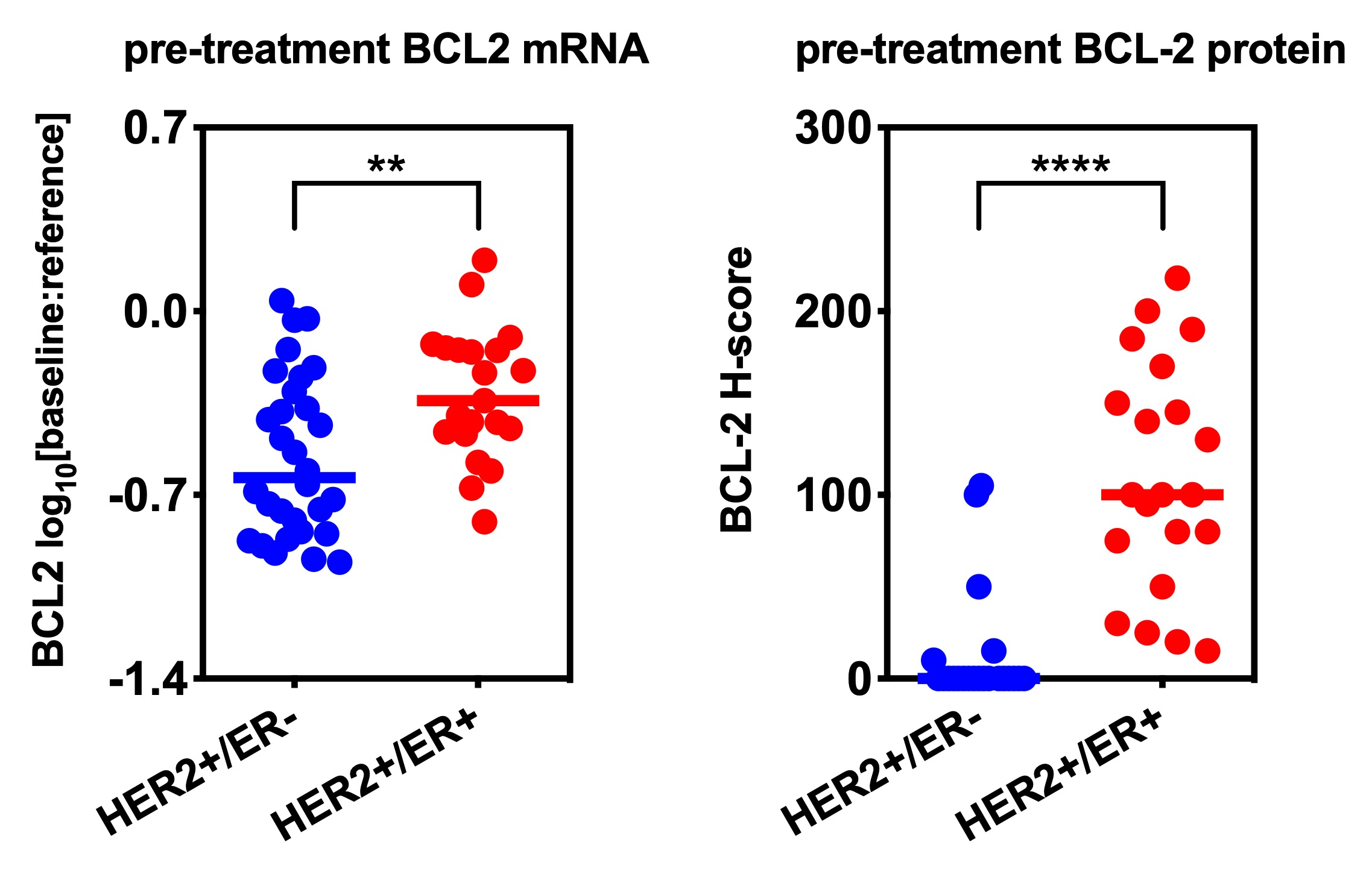

Supplement: S1 Fig — Each plot compares baseline (pre-treatment) HER2+/ER- (pre-treatment ER H = 0) and HER2+/ER+ (pre-treatment ER H > 0) tumors. Statistical comparisons of BCL2 mRNA utilized Significance Analysis of Microarrays (SAM) analyses, FDR-p = 0.00319 (**). Statistical comparisons of BCL-2 protein utilized Mann-Whitney two-tailed test, p < 0.0001 (****). Each line represents the median. (JPG) [file pone.0251163.s001.jpg]

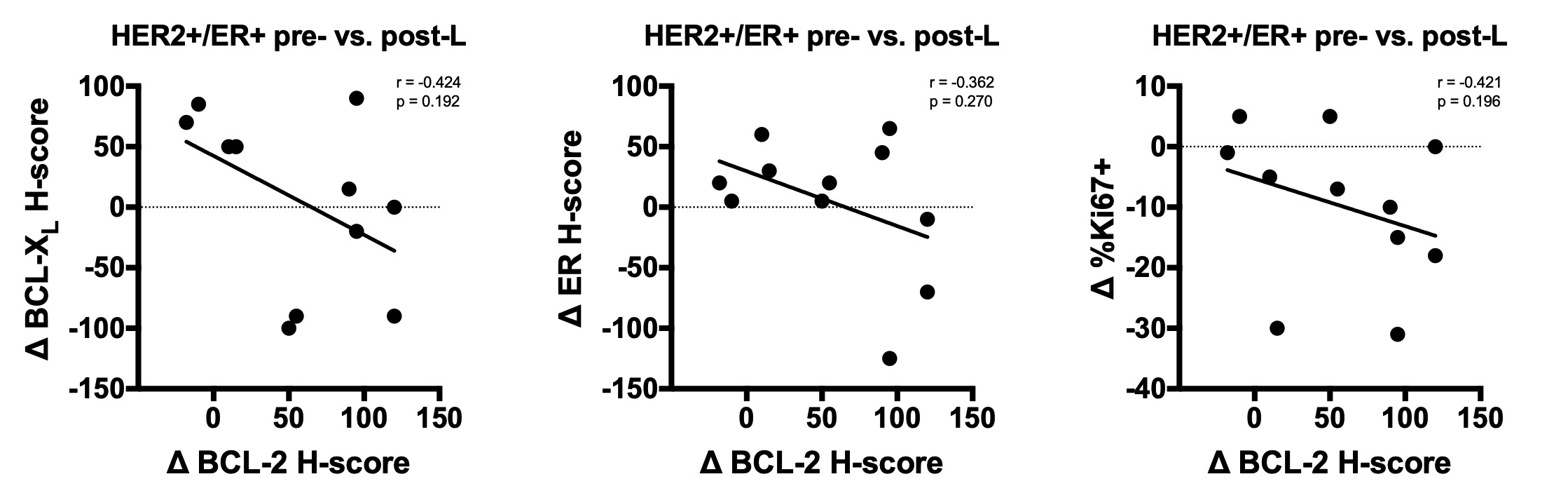

Supplement: S2 Fig — Each graph compares BCL-2 and BCL-XL, ER or Ki67 alterations (Δ = Fig 4 post-value–Fig 4 pre-value). The Spearman correlation [r] and associated p-value [p] is indicated for each comparison. 95% CI: BCL-2 and BCL-XL [-0.8231, 0.2548], ER [-0.7980, 0.3219] or Ki67 [-0.8218, 0.2585]. (JPG) [file pone.0251163.s002.jpg]
